# Chemical Observations on the Epidermis

**Published:** 1799-05

**Authors:** I. A. Chaptal


### *Chemical Observations on the Epidermis:—By* I. A. Chaptal.

THE human skin is perhaps that which exhibits the Epidermis most conspicuously, and easily to be removed: upon it have been made the experiments which I now proceed to detail.

The human skin becomes hardened by means of hot water, and separates into two distinct parts, the epidermis and cutis; the latter of which has the consistence of soft cartilage.

The continued action of hot water will at last dissolve the cutis, and yet not sensibly attack the epidermis.

Alkohol

Alkohol topically applied to the epidermis, makes no impreſſion upon it: cauſtic alkali diſſolves it; lime produces the ſame effect, but more ſlowly.

There is therefore an analogy between the external covering of the human body and that which covers ſilk-pods.

From theſe principles we may draw ſome conſequences that may be eaſily applied to the operations of tanning.

1. If a ſkin, covered with its epidermis, be plunged into an infuſion of tan, the tan will only penetrate the fleſh ſide; the other ſide being defended by the epidermis, which is not ſuſceptible of the tanning principle.

2. When the epidermis is taken off, by the operation of peeling, then the tan penetrates the two ſurfaces of the ſkin.

3. The lime generally uſed in this proceſs, appears only to act in diſſolving the epidermis. Lime-water has more action than lime not diſſolved; but, its effect ceaſes the moment the lime held in ſolution is combined: hence the neceſſity of renewing the lime-water, to accompliſh the taking off the ſkin.

